# Health Communication through Positive and Solidarity Messages Amid the COVID-19 Pandemic: Automated Content Analysis of Facebook Uses

**DOI:** 10.3390/ijerph19106159

**Published:** 2022-05-19

**Authors:** Angela Chang, Xuechang Xian, Matthew Tingchi Liu, Xinshu Zhao

**Affiliations:** 1 Department of Communication, Faculty of Social Sciences, University of Macau, Macao, China; yb97322@um.edu.mo (X.X.); xszhao@um.edu.mo (X.Z.); 2 Institute of Communication and Health, Lugano University, 6900 Lugano, Switzerland; 3 Department of Communication, Zhaoqing University, Zhaoqing 526060, China; 4 Department of Management and Marketing, Faculty of Business Administration, University of Macau, Macao, China; matthewl@um.edu.mo

**Keywords:** COVID-19, Facebook, positive psychology, solidarity, anti-epidemic, semantic analysis, natural language processing, automated content analysis

## Abstract

The COVID-19 outbreak has caused significant stress in our lives, which potentially increases frustration, fear, and resentful emotions. Managing stress is complex, but helps to alleviate negative psychological effects. In order to understand how the public coped with stress during the COVID-19 pandemic, we used Macao as a case study and collected 104,827 COVID-19 related posts from Facebook through data mining, from 1 January to 31 December 2020. Divominer, a big-data analysis tool supported by computational algorithm, was employed to identify themes and facilitate machine coding and analysis. A total of 60,875 positive messages were identified, with 24,790 covering positive psychological themes, such as “anti-epidemic”, “solidarity”, “hope”, “gratitude”, “optimism”, and “grit”. Messages that mentioned “anti-epidemic”, “solidarity”, and “hope” were the most prevalent, while different crisis stages, key themes and media elements had various impacts on public involvement. To the best of our knowledge, this is the first-ever study in the Chinese context that uses social media to clarify the awareness of solidarity. Positive messages are needed to empower social media users to shoulder their shared responsibility to tackle the crisis. The findings provide insights into users’ needs for improving their subjective well-being to mitigate the negative psychological impact of the pandemic.

## 1. Introduction and Literature Review

### 1.1. COVID-19 Infodemic and Positive Communication in Macao

The highly transmissible and contagious nature of the SARS-CoV-2 has posed a threat to public health and safety. The World Health Organization declared coronavirus 2019 (COVID-19) a public health emergency of international concern in March 2020 [1]. To date, the ongoing pandemic has led to over 270 million infections and approximately 5 million deaths [2].

During the pandemic, social media platforms have been increasingly used to convey impactful messages to the masses [3,4]. Although it is considered an effective form of communication for raising public awareness, negative messages shared across social media can in turn fuel negative emotions, such as sadness, discrimination, and hatred [5,6]. This may subsequently lead to harmful behavioral tendencies, such as blaming others, engaging in violence, and contemplating or attempting suicide, all of which have been particularly evident during the COVID-19 pandemic [7]. 

Extant literature has examined the impact of positive affect in communication [8,9]. As a general dimension of mood, positive affect reflects the subjective well-being of individuals when engaging with the environment. High positive affect among the public indicates more subjective well-being and engagement [9]. Therefore, to alleviate negative impact caused by the COVID-19 pandemic, positive affect should be maintained. 

As one of the world’s most densely populated cities that heavily relies on tourism and gambling industries [10], Macao has recorded only 77 confirmed COVID-19 cases and zero deaths as of 10 November 2021 [11]. The local government’s efforts to contain the spread of the virus have earned nationwide praise [12,13]. Several preventive policies, such as social distancing, self-quarantine, and mask-wearing, have been enacted and have achieved satisfactory outcomes. Meanwhile, laypeople’s contributions are evident in their commendation of the government’s and frontline workers’ efforts and in uplifting messages on social media. Phrases and hashtags, such as “we stand in solidarity” and “fight together against the COVID-19”, were frequently disseminated by Macao citizens online, creating a sense of togetherness through collaborative efforts in fighting the pandemic.

### 1.2. Positive Psychology Attributes

Individuals’ subjective well-being is associated with positive psychology attributes, such as solidarity, hope, and gratitude [14,15,16]. Solidarity refers to unity, or collective actions, or mutual support within a group, which is formed through common interests to improve outcomes in all aspects of life [17]. Prior research has found that shared threat experience increases identification with those suffering the same fate, which leads to increased solidarity [18]. This mechanism is also held true in the context of the COVID-19 crisis, where solidarity among individuals was found to be more profound than that within other previous crises [19,20]. Given that a viable way to contain the disease is cooperation at individual, national and global levels, the positive response of solidarity can thus be considered a key resource in coping with the crisis by facilitating internal or external cooperation [18,21]. For example, with increased social bonding, solidarity was found effective in promoting people’s willingness to support for the COVID-19 infection control measures enforced by the government [22].

Hope is considered a powerful source of motivation to develop resilience and cope with stressful situations by alleviating negative psychological factors, such as distress, anxiety, and depression [15,23]. Individuals with a higher level of hope tend to have lower levels of depressive symptoms and anxiety [24]. In other words, hope enables one to adapt to a stressful situation, such as the COVID-19 crisis [25]. 

Optimism is generally defined as an inclination toward expecting a positive, favorable, or desirable outcome. It provides a positive outlook for people to make efforts when facing difficulties [26]. Optimism mediates spiritual well-being’s effect on negative psychological emotions, and a higher level of optimism leads to lower levels of mental distress, anxiety, and depressive symptoms [27,28]. Therefore, optimism is considered a protective factor to mitigate the impact of stress on psychological health and essential for meaning-centered intervention [29]. 

Gratitude is a positive response to a good outcome of another individual’s efforts. It encourages prosocial responses among the beneficiaries and is associated with lower depressive symptoms, burnout, and suicide risk in a non-crisis context [16,30,31]. In a crisis context, gratitude is a key driver of adaptation to crises, such as COVID-19 [32].

In positive psychology, grit is defined as perseverance and passion toward one’s pursuit of long-term goals [33]. Studies have demonstrated that grit can predict psychological well-being, such as higher positive emotions, and positively affect mental health and social performance [34,35]. Grit has also been shown to buffer mental distress caused by the fear of COVID-19 [36].

In light of these findings, solidarity, hope, optimism, gratitude, and grit, which are rooted in positive psychological theories, can be considered coping resources in response to the pandemic. While consistently sharing messages about positive coping have been employed as a strategy to inspire the public, studies of positive coping messages have been more focused on discourse analysis from a qualitative perspective [4,37], where researcher bias is inevitable. In addition, studies of negative information, such as discriminatory and stigmatized information linked to COVID-19, have received much attention [5,38]. However, there is a limited understanding of the mechanism of the dissemination of positive coping information, which helps mitigate social anxiety and promote society’s well-being. This indicates the need for more empirical studies of positive responses in the context of health crises. Therefore, in this study, we aimed to examine the corpus of Facebook posts to understand how positive coping messages were propagated and to determine the level of public engagement with positive messages during the COVID-19 outbreak in Macao. 

The present study attempted to answer the following research questions:

RQ1: How much discussion was devoted to the positive coping of COVID-19 pandemic on Facebook? 

RQ2: What types of themes were presented most in the Facebook postings examined?

RQ3: What were the associations between posting themes and various crisis stages?

RQ4: What were the associations between content types, multimedia elements, posting themes, crisis stages, and the level of public engagement? 

### 1.3. Measurements 

Public response and user engagement in mitigating a negative outcome of a crisis interact with different crisis stages [39]. These stages constitute the crisis lifecycle, which follows an evolutionary pattern from precursor to resolution. In this study, a framework was adopted for the association between positive response and public engagement based on the crisis lifecycle model proposed by Fink [40]. 

A crisis is a dynamic and cycling process, which has been recognized since 1980 [41]. Several crisis lifecycle models have been developed for studying crisis management [42,43,44]. Considering that the COVID-19 pandemic is an ongoing crisis with a complicated process of evolution, and that Fink’s model provides a detailed and clear understanding of a crisis [45], the model thus served as a theoretical basis for our study.

Fink’s model describes four stages of a crisis: prodromal, acute, chronic, and resolution [40]. Specifically, the prodromal stage refers to the preparation stage, which highlights the emergent clues of a potential crisis and the beginning of taking preventive measures. It plays a crucial role in determining whether the stakeholders are moving toward a crisis or wellness. Macao has been on high alert since the COVID-19 outbreak in Wuhan, China. The local government has taken preemptive measures to respond to the imminent threat of the health crisis [46]. The first stage, thus, corresponds to the starting point of the COVID-19 outbreak in Wuhan at the beginning of January 2020 [5]. Following the prodromal stage (1–21 January 2020), the acute stage is triggered by an event that indicates the beginning of a crisis [39,40]. During this period, damage occurs at a tremendous rate with considerable fatalities, property losses, and security losses. These correspond to the period between 22 January and 19 April 2020, when the pandemic broke out in Macao [11]. The chronic stage highlights the lasting effect of the crisis. This corresponds to the period between 20 April and 31 December 2020, when the outbreak in Macao had been brought under control [47]. The last stage is the resolution stage, which indicates that the crisis has ceased. Considering the current threat of the pandemic in Macao, we did not include this stage in our study.

## 2. Methods

The workflow of this study mainly comprised four components: (1) data crawling and screening; (2) Word2vec embedding for positive keywords development; (3) computational-assisted data processing; and (4) statistical data analysis and results-reports visualizations. 

### 2.1. Automatic Content Analysis

Automatic content analysis gauges digital traces and measures online users’ behavior unobtrusively. Several studies have been employing this method to study online health communication by discovering communication trends and patterns from a large amount of unstructured text corpus (e.g., [5,48]). DivoMiner, a text-mining and automatic content analysis platform supported by machine learning algorithms, was then employed in this study specifically for identifying Chinese language texts. The computational platform combined automatic content analysis while considering traditional content analysis procedures. DivoMiner provides functions, such as data processing and filtering, keywords establishing and screening, inter-coder reliability testing, machine/manual coding, and quality monitoring. Several studies have applied it to learn about health crises, disease news coverage, policy implementation, and other topics (e.g., [49,50]). In the present study, the DivoMiner platform was employed for the following: (1) acquire preliminary data on COVID-19-related posts in Chinese language using predesigned search terms; (2) conduct word segmentation, stop word removal of the crawled data and conduct relevant data screening for data preprocessing; (3) determine positive messages using sentiment polarity analysis; and (4) combine automated content analysis and manual verification with acceptable reliability. 

### 2.2. Word2vec Word Embedding Technique

Word2vec word embedding is a natural language processing technique that maps each word into a vector space, wherein words that have similar contexts in the corpus are juxtaposed [51]. Therefore, it enables the detection of synonymous words by learning word associations from a large corpus of text, using neural network modes. This technique has been applied to many fields, such as community detection [51], lexicon construction [52], and named entity recognition [53]. In this study, Word2vec word embedding was applied to facilitate keyword development for machine coding.

### 2.3. Sentiment Polarity Analysis

Sentiment polarity analysis was also performed to identify posts with positive sentiment through DivoMiner [5,54]. The big data analysis platform enables the classification of sentiment polarity within the collected data by incorporating the most common sentiment lexicons, such as the LIWC lexicon, DLUT lexicon, and CNKI lexicon, while considering factors such as exclamation marks and negative words [5,55]. The platform matches the words in the posts and those in the lexicons to determine the sentiment polarity of each post. In this study, the positive messages identified were used as a sub-corpus for automated coding.

### 2.4. Semantic Network Analysis

For correlation and total effects analysis, a semantic network analysis (SNA) was performed to examine co-occurrences based on their paired presence within a text corpus [56]. Networks are created by linking pairs of terms based on their co-occurrence. For example, a pair is considered to co-occur if both terms appear in a particular message. SNA is useful to analyze big data, such as social media posts, by revealing the main structure in the text. The UCINET 6.730 software (Analytic Technologies, Lexington, KY, USA) was employed to calculate and plot a semantic network wherein each concept of positive messages was treated as a node and the co-occurrence of a pair of concepts as a link. The degree centrality (DC), which measures a concept’s centrality and power potential, was calculated to determine the most prominent concept. The link strength was visualized in edge width to distinguish different levels of co-occurrence within the network. 

### 2.5. Data Collection and Verification

One year of data, from 1 January 2020 to 31 December 2020, were collected to ensure the appropriateness of the public opinion reflected. Facebook was used as the data source as it is one of the largest social networking sites around the world with roughly 2.91 billion monthly active users as of the third quarter of 2021 [57]. Facebook features a relatively free flow of information among users compared to traditional media. In Macao, 94% of the residents aged 18–34 years engage in their online activities (e.g., online discussion and information acquiring) through Facebook [58]. 

Facebook posts and the corresponding data on public engagement were retrieved through the DivoMiner platform. As in a previous study [59], this study used the number of “likes” as an indicator of public engagement, as it is considered an ideal metric that represents public attitude within a large sample of various sources. 

After extracting all the COVID-19-related messages from Facebook, messages that were duplicated or irrelevant to the COVID-19 crisis discussion were removed. A total of 104,827 valid posts were included in our dataset for analysis, after eliminating 2385 posts. To ensure the validity and reliability of the machine-generated output, a manual verification approach was used. Two trained coders, who are native Cantonese speakers with a background in communication, were trained for 12 h. They then checked a sample of 500 posts as a pilot test for the sentiment and content analysis. The intercoder reliability, measured by Krippendorff’s alpha, ranges from 0.82–0.88, which are exceedingly high scores given the uneven distributions [60,61]. The consistency of the results of manual coding and machine coding reached an acceptable level of 84%, as indicated by previous studies (e.g., [5,49,55]). 

### 2.6. Keywords Developed for the Automated Coding

The concepts of the examined messages were primarily adapted from the aforementioned literature [14,15,16,26,33]. However, a preliminary investigation of Facebook posts revealed that the anti-epidemic narrative was prominent in public communication. Considering the importance of the general public’s adherence to COVID-19 control measures, messages with emphasis of the goal of anti-epidemic can also be included as part of the public’s positive coping resource responding to the pandemic. Thus, our codebook consisted of the following themes: (1) solidarity; (2) anti-epidemic; (3) gratitude; (4) hope; (5) optimism; and (6) grit. The list of themes, definitions, and examples is provided in Supplementary File S1.

For preliminary data acquisition, COVID-19-related keywords and terms used to query the data source were determined based on an existing study and an official document [5,62]. Keywords for the automated coding were developed using word2vec word embedding toolkit imported from the Python 3.7.4 Gensim module [63]. As a natural language processing library, Gensim functions include extracting semantic features from the corpus and converting words to word vectors. First, we conducted model training using all the collected data (*n* = 104,827) as the text corpus. The continuous bag-of-words architecture was applied with negative sampling. Based on a previous study [53], the dimension of embedding was determined to be 50. 

Second, the cosine similarity calculation method was applied to indicate the level of semantic similarities between keywords with larger values indicating more relevance between the two vectors and smaller values indicating less similarity [63]. Keywords above a similarity threshold of 0.46 were selected, which, in our case, reflected a relatively higher accuracy of data. Considering the complex nature of the Chinese language, the results were checked and corrected. We screened the extracted keywords based on their relevance to the context, and we referred to a Chinese thesaurus to annotate the synonyms within the sample. The screening and annotation results were then synthesized for developing a codebook for machine coding. The list of keywords for data acquisition and machine coding is presented in Supplementary File S2.

### 2.7. Statistical Analysis

SPSS (version 23, IBM Inc., Chicago, IL, USA) was used for statistical analysis in this study. Due to the overdispersion of the number of likes, we used median, rather than mean, to describe the central tendency of the data, based on empirical studies [39,64]. A chi-square test for independence was used to test the significance of the association between crisis stages and posting themes. A post hoc pairwise comparison test was performed for the significant chi-square outcome to compare each pair of crisis stages. Testing of all pairwise comparisons was adjusted using Bonferroni correction.

The nonparametric Kruskal–Wallis test was applied to assess public engagement by different crisis stages. The Mann–Whitney U test was used to compare public engagement between Facebook posts containing specific themes and those that did not. 

A negative binomial regression analysis was performed to examine the associations between predictors (content types, multimedia element, posting themes, and crisis stages) and public engagement, owing to the positively skewed distribution of likes. A significance level of 0.05 was applied to all statistical tests in this study. 

## 3. Results

### 3.1. Basic Findings

Of the 104,827 posts related to the COVID-19 crisis, 60,875 posts were identified as positive messages. Furthermore, the positive themes examined were identified in 24,790 positive messages collected after machine coding. These messages were posted by 1573 unique Facebook accounts and had been reposted 135,562 times; the cumulative number of comments and likes were 161,598 and 710,089, respectively. 

The most popular positive message that received 10,335 likes was posted on 9 February 2020 by a local online news media called Love Macao. The post was a video shared from Douyin (the Chinese version of TikTok); the video showed a real moving story about medical workers working at the frontline to manage COVID-19. 

**Figure 1:**
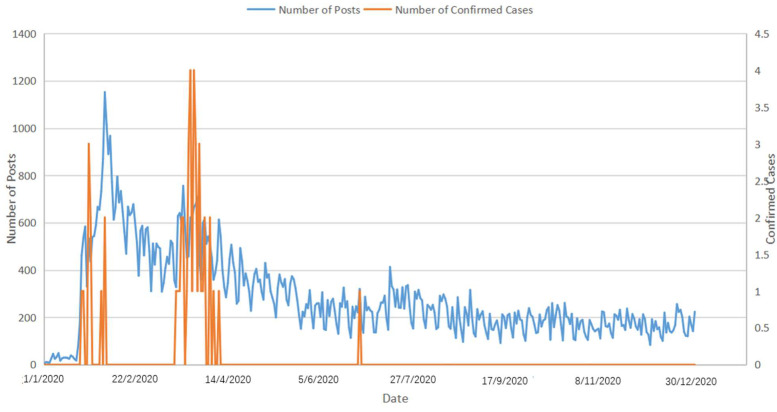
Number of collected posts compared with infectious cases (1 January to 31 December 2020).

Overall, the daily volume of online COVID-19 discussion ranged from 3 to 1152 posts, with a mean value of 286 (SD = 185). Specifically, 736 COVID-19-related messages were posted during the prodromal stage, when the pandemic spread in Wuhan and other places in China, but not yet in Macao (35 posts per day on average). Figure 1 shows a plot of Facebook posts pertinent to COVID-19 versus the number of confirmed cases in Macao (see Figure 1).

**Figure 2:**
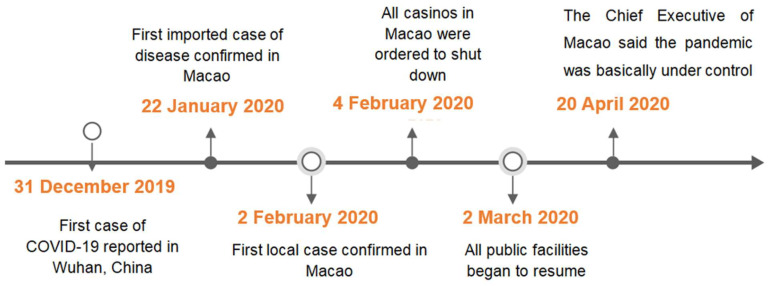
Timeline of the key events of the COVID-19 pandemic in Macao (1 January to 31 December 2020).

Between 22 January and 19 April 2020, Facebook discussions around the pandemic consistently emerged with the rising number of reported cases (*n* = 48,679), and an average number of 547 posts per day were made. This period has witnessed the highest frequency of discussion over the 12 months. Online discussions on COVID-19 peaked at over 1000 posts on 4 February 2020, when all casinos and other entertainment facilities in Macao were ordered to shut down [46]. On 20 April 2020, the Chief Executive announced that the pandemic was under control. Following this, the government lifted restrictions and resumed all public facilities. Subsequently, discussions on COVID-19 began to diminish and plateaued by the end of the year, with an average of 217 posts per day (*n* = 55,412). A timeline depicting the key events of the COVID-19 pandemic in Macao is presented in Figure 2. 

### 3.2. Interconnection of Positive Concepts by SNA

Of the 24,790 Facebook posts examined, over 50% comprised anti-epidemic and related terms (*n* = 14,219; 57.4%); 37.6% mentioned solidarity (*n* = 9, 324); and 31.1% contained messages that reflected hope (*n* = 7698), followed by gratitude (*n* = 5186, 20.8%), optimism (*n* = 1626, 6.6%), and grit (*n* = 715, 2.9%). 

**Figure 3:**
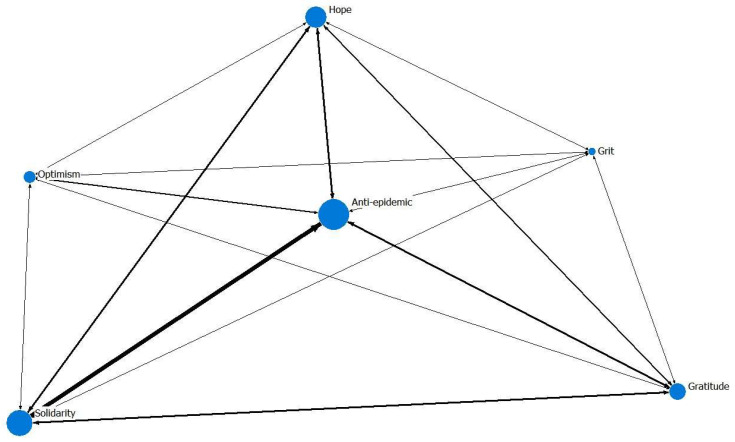
Semantic network depicting the association among different concepts based on co-occurrence.

The Freeman Degree Centrality measurement showed a relatively low concentration of the network (DC = 0.2517). Specifically, the concept of anti-epidemic had the highest degree centrality (nDegree = 0.412), followed by solidarity (nDegree = 0.408), gratitude (nDegree = 0.257), hope (nDegree = 0.244), optimism (nDegree = 0.092), and grit (nDegree = 0.053). The pairs of concepts that were highly associated were anti-epidemic and solidarity (*n* = 5327), anti-epidemic and gratitude (*n* = 4426), and hope and solidarity (*n* = 2308); meanwhile, there was very little interaction between optimism and grit (*n* = 49). Figure 3 shows the strength of the concepts associated with each other in an undirected semantic network. 

### 3.3. Messages with Positive Themes across Different Crisis Stages 

Messages mentioning optimism and grit accounted for a relatively low proportion of all posts across the crisis stages (χ_2_^2^ = 168.565, *p* < 0.001; χ_2_^2^ = 165.700, *p* < 0.001). Messages mentioning solidarity were the most salient at the prodromal stage (55%), while its proportion decreased at the acute (41%) and chronic (35%) stages (χ_2_^2^ = 108.615, *p* < 0.001). Conversely, the percentage of anti-epidemic posts was less noticeable at the prodromal stage (25%), but the proportion increased significantly and peaked at the acute (65%) stage (χ_2_^2^ = 591.108, *p* < 0.001). The number of messages related to hope was higher at the prodromal (39%) and chronic stages (37%) than at the acute (24%) stage (χ_2_^2^ = 509.099, *p* < 0.001). Lastly, the frequency of messages that mentioned gratitude substantially increased from 14% to 22% at the chronic stage (χ_2_^2^ = 10.821, *p* = 0.004). The message themes across the crisis stages are summarized in Table 1 and Supplementary File S3.

### 3.4. Public Engagement with Positive Postings

The result of Kruskal–Wallis H test showed significant differences in the frequencies of likes (χ_2_^2^ = 557.278, *p* < 0.001) among different crisis stages. Post hoc pairwise comparisons (see Supplementary File S4) showed no significant difference in the frequencies of likes between the prodromal and chronic stages (Z = −543.086; *p* = 1.000).

For the complete sample, the mean number of likes was 28.6 (SD = 142.651). As the continuous variable was positively skewed (skewness = 35.089), the median was used as an alternative to better describe the central tendency of the number of likes (Mdn = 5.0). Specifically, our results revealed that users interact most with positive messages during the acute stage, with the highest median number of likes (Mdn = 7.0) but a low mean number of likes at this stage (M = 38.0). Public engagement levels (measured by the number of likes) across different crisis stages and posting themes are outlined in Table 2. 

The Mann–Whitney U tests further showed that frequencies of likes (Z = −2.385; *p* = 0.017) at the prodromal stage were significantly lower for messages that advocated solidarity than those that did not. Conversely, during the acute stage, messages mentioning gratitude (Z = −7.721; *p* < 0.001), hope (Z = −2.366; *p* = 0.018), and optimism (Z = −7.040; *p* < 0.001) received more likes than those that did not. Regarding messages posted during the chronic stage, those expressing solidarity (Z = −2.779; *p* = 0.005), hope (Z = −6.322; *p* < 0.001), gratitude (Z = −9.430; *p* < 0.001), and optimism (Z = −5.371; *p* < 0.001) attracted more likes than those that did not, while messages that highlighted anti-epidemic received less response in terms of likes than those that did not (Z = −12.368; *p* < 0.001). Public engagement with specific themes at different stages are presented in Table 3. 

A negative binomial regression analysis was performed with the outcomes summarized in Table 4. This model has been optimized compared with the null model, which treated factors as a set to predict the public engagement level. The Wald test showed that all the predictors were significant (*p* < 0.001), except for the association between solidarity and the number of likes (*p* = 0.295). 

Our results revealed that posting albums (IRR = 1.735; *p* < 0.001), hyperlinks (IRR = 1.289; *p* < 0.001), photographs (IRR = 1.662; *p* < 0.001), statuses (IRR = 1.245; *p* < 0.001), and videos (IRR = 2.004; *p* < 0.001) were all significantly associated with higher number of likes, whereas the use of notes (IRR = 0.754; *p* = 0.017) was significantly associated with a lower number of likes. 

Regarding the impact of positive concepts on public engagement, messages mentioning gratitude (IRR = 1.573; *p* < 0.001), hope (IRR = 1.190; *p* < 0.001), and optimism (IRR = 1.522; *p* < 0.001) were significantly associated with higher engagement levels. In contrast, those that mentioned grit (IRR = 0.870; *p* < 0.001) and anti-epidemic sentiments (IRR = 0.897; *p* < 0.001) were significantly associated with a lower number of likes.

Messages posted during all crisis stages showed significant associations with higher engagement levels (prodromal stage: IRR = 5.093, *p* < 0.001; Acute stage: IRR = 1.719, *p* < 0.001; IRR = 1.149, *p* < 0.001). 

## 4. Discussion

### 4.1. Principal Findings

Unpleasant emotional states, such as anxiety or fear, can motivate an individual to change oneself or their situation to increase the possibility of success or to avoid danger. Unpleasant emotions, despite their negative effects, have the potential to keep us safe by motivating us to improve our lifestyles. In comparison, positive emotional states, such as hope, solidarity, and gratitude have several benefits in building our resilience, broadening our awareness, and letting us see more options for problem solving. Solidarity, for instance, has been linked to collaborative efforts and collective success, as well as beneficial outcomes for strengthening mental health. Unchecked solidarity, however, can increase the risks of fanaticism behaviors and bring about unpleasant emotions that come with it. Nonetheless, positive affect has an important role in helping the public overcome the hardships caused by the pandemic.

This present study therefore investigated how Macao’s citizens coped with stress using Facebook during the COVID-19 epidemic. The results showed that positive affect was the dominant emotional aspect across Facebook posts during the COVID-19 outbreak. Communication with positive messages was frequently used as a means of positive coping in response to the pandemic. In turn, it influences their recipients’ cognitive and emotional responses via emotional appeal. Persuasive messages, such as “Together, we fight the pandemic” and “Sustain together through the difficult times”, elicited solidarity among recipients and increase communal awareness. However, our finding on the predominance of positive affect in response to the crisis may challenge fatalistic beliefs about the COVID-19 pandemic. Similar to non-communicable diseases, coronavirus has often been accompanied by mortality in news media’s portrayal, eliciting heated discussion and perception of gloomy fatalism [5,54,55]. Studies have concluded that fatalism undermined individuals’ intention to comply with COVID-19 prevention measures, including social distancing and hand washing [65,66]. Given the traditional Chinese philosophies of Confucianism and Buddhism, which have influenced one’s attribution of life events to fate [67,68], Chinese people are more inclined to be fatalistic when faced with misfortunes caused by the COVID-19 epidemic [69]. Thus, messages about positive coping in response to the COVID-19 epidemic could challenge fatalistic thinking about the crisis and increase coping efforts.

All the positive concepts were presented at different levels. Specifically, anti-epidemic was the most common concept appearing in the positive messages. Within the semantic network, the concept of anti-epidemic interacted most frequently with other concepts. A high degree of centrality of this concept can be interpreted in terms of the active state of the node for interacting with other positive concepts. The concepts of solidarity and anti-epidemic significantly appeared together in most messages, with examples such as “Let’s work together to fight the epidemic” and “As long as the whole society is united and adheres to the core values of our times, we will surely defeat the epidemic”. This is not surprising, given that the COVID-19 disease is a global health crisis of our time, which has presented huge challenges to the social and economic systems. Combating the unprecedented pandemic and ending the alarming situation demand collective efforts and cooperation within the civil community.

Hope was the third most frequent concept in the examined posts with examples such as “Hope the epidemic will end soon, and everyone is safe and healthy” and “I wish the epidemic can be brought under control as soon as possible and the anti-COVID-19 drugs will be in place soon”. This is reasonable as studies have shown that people seek hopeful messages when faced with a stressful and fearful situation, such as the COVID-19 pandemic [70,71]. Thus, expressing hope can be understood as the simplest way for individuals to alleviate negative affect, such as stress, anxiety, and uncertainty about the future, thereby improving their subjective well-being. Furthermore, the concept of hope was often associated with solidarity and anti-epidemic in the examined messages, suggesting that many hope-related messages on Facebook conveyed the belief that the crisis can be overcome by reinforcing solidarity and collaboration, at least to some extent. Such messages provided a powerful source of motivation for recipients to adapt to the threat of COVID-19. 

This study clarified the distribution of positive messages across different crisis stages. Overall, the number of positive messages examined (*n* = 24, 790) was relatively low at the prodromal stage (2.1 posts per day on average), whereas this figure increased dramatically at the acute stage when the pandemic broke out in the region (134 posts per day on average) and decreased at the chronic stage (51 posts per day on average). The increase in the number of posts might be because of information anxiety, which, in turn, encourages more positive messages [72].

During the prodromal stage, solidarity-related words were used more frequently in positive communication. This was followed by the concepts of hope, anti-epidemic, and gratitude, while those of optimism and grit were barely mentioned. During the acute stage, the proportion of anti-epidemic messages was higher than that of messages emphasizing solidarity; it became the most prominent concept among all positive ones, indicating a strong desire to contain the spread of COVID-19 in Macao. Further, messages mentioning gratitude, optimism, and grit increased substantially during this stage.

As the crisis developed into the chronic stage, messages with anti-epidemic terms continued to prevail. This could be relevant to the chronic nature of the COVID-19 pandemic that created a “new normal” of coping with the pandemic [39]. Furthermore, a considerable percentage of messages at this stage included hope and solidarity. The distribution of messages across different stages may indicate a change in the general public’s psychological status, which is associated with the progression of the pandemic [73]. 

The results of public engagement showed that the level of “likes” was largely consistent with the progression of the crisis. Messages expressing anti-epidemic concept, solidarity, hope, gratitude, optimism, and grit elicited more “likes” during the acute stage than during other stages. This is not surprising given the outbreak of the disease and the severity of stress at the acute stage, which might explain why positive thinking was much more favored. Specifically, messages that mentioned hope and gratitude obtained a higher level of engagement at the prodromal stage, which may indicate that people were inclined to expect a good outcome and to demonstrate their prosocial behavior [16,23]. Messages mentioning gratitude and optimism during the later stages elicited more public engagement than other positive concepts during the acute and chronic stages. Based on these findings, we may infer that as the pandemic broke out and progressed in Macao, people were inclined to improve their subjective well-being [71] and be prosocial to adapt to the crisis [32].

The positive concepts, except for solidarity, expressed in the posts were significantly associated with public engagement. Posts mentioning gratitude, hope, and optimism received higher engagement levels, suggesting that users prefer these types of information during a health crisis, to improve their mental state. Accordingly, it may be essential for crisis managers, health communicators, and even regulators to explicitly explore the concepts of gratitude, hope, and optimism that not only emphasize “seeing the positive” but also help cope with social deprivation, uncertainties, difficulties, and stressful situations [23,74,75]. Communicators should be encouraged to use language that reinforces gratitude, hope, and optimism during such situations, and specific interventions should be developed to mitigate the pandemic’s negative psychological impact on the general population.

The positive concepts of anti-epidemic and grit require efforts from people to overcome difficulties. While the terms “anti-epidemic” and “grit” have been emphasized to motivate people to positively respond to the COVID-19 pandemic, our findings indicate that messages with these concepts are less likely to receive public engagement. A potential explanation for these findings could be that the low infection rate in Macao led to a lower perception of risk among people, which subsequently influenced their response to such messages. 

### 4.2. Comparison with Prior Work

In this study, we found that Facebook is an effective channel to share, communicate, and disseminate information during the COVID-19 pandemic in Macao. Overall, Facebook users’ responses to COVID-19 to some extent aligned with the progression of the pandemic in Macao. Other studies have also reported similar findings during the COVID-19 outbreak [39,76]. 

We also identified several factors associated with public engagement during the pandemic. First, the presence of the media elements, such as hyperlinks and statuses, was positively associated with the number of likes. This is inconsistent with a previous study, which showed that the use of hyperlinks might reduce public engagement because it requires additional clicking action from the recipients [77]. This discrepancy could be due to the different natures of the messages, as most studies that found a negative effect examined messages without considering the factor of emotional valence. Second, our result that the inclusion of multimedia content (e.g., albums, photographs, and videos) is positively associated with public engagement supports previous studies, which found positive effects of media on public engagement [64,78].

### 4.3. Limitations 

This study has several limitations. First, the examination of positive communication during the COVID-19 pandemic only focused on one geographical area of China. Since different regions may vary significantly in terms of social, cultural, and political characteristics, as well as in terms of their social media usage, various communication patterns must be developed. Further studies on positive communication during the COVID-19 in different regions such as mainland China, Hong Kong, Taiwan, or even western countries should be considered. Second, since the pandemic is still at large, this present study only examined the prodromal, acute, and chronic stages but ignored an inquiry into the ongoing stages of resolution. To provide a full picture of positive communication on Facebook during the COVID-19 pandemic, the scope of analysis should be expanded in a future study. Another important limitation of this study comes from capturing users’ data on Facebook. Despite the fact that we extracted data from Facebook to inquire into users’ online interactions, further information, such as individual’s identity, age, social class, gender or other relevant data remained unknown. Having such information is useful for identifying diverse behavioral patterns depending on different individual and socio-demographic contexts when managing the pandemic. It can also provide us more insight into the underlying mechanism of the fostering of solidarity among different types of populations. Therefore, further attention may need to be paid in the future to address this issue.

## 5. Conclusions

The COVID-19 outbreak, and its subsequent effects have increased the risk of poor mental health outcomes among the general population, which is a significant challenge for tackling the pandemic. Thus, creating a positive atmosphere in crisis communication is imperative. This study focused on Facebook users’ online responses during the COVID-19 outbreak in Macao from a positive perspective to explore communication patterns and provide insights into effective communication strategies development. Our results found significant differences in posting themes and public engagement across different crisis stages. Anti-epidemic and solidarity were the most prevalent concepts, although these received less public engagement than other concepts such as gratitude and optimism.

This study serves as a starting point for further research into the role of positive messages in crisis communication. The case of Macao in our study provides several implications for crisis experts, health authorities, and governments to manage a challenging situation through communication. First, circulating information on social media helps reach diverse populations. Sharing positive messages through Facebook to various social circles may also have influenced individual and social cognition. Second, using positive phrases of unity in online communication may not be enough to elicit public response to solidarity. Messages that enable citizens to realize their common problems and empower themselves to shoulder their shared responsibilities in overcoming the difficulties may also need to be considered. Third, communication experts and crisis managers should also consider the general public’s mental needs to improve their subjective well-being, which can not only increase public engagement, but also mitigate negative psychological effects during a crisis.

## Figures and Tables

**Table 1:** Themes of posting across different crisis stages in Macao (1 January to 31 December 2020).

Theme	Prodromal Stage (*n* = 44)	Acute Stage (*n* = 11,929)	Chronic Stage (*n* = 12,817)	Overall (*n* = 24,790)
Anti-epidemic	11 (25)	7779 (65)	6429 (50)	14,219 (57)
Solidarity	24 (55)	4870 (41)	4430 (35)	9324 (38)
Hope	17 (39)	2883 (24)	4798 (37)	7698 (31)
Gratitude	6 (14)	2399 (20)	2781 (22)	5186 (21)
Optimism	1 (2)	1035 (9)	590 (5)	1626 (7)
Grit	0 (0)	176 (1)	539 (4)	715 (3)

Note: A post can belong to more than one theme. Values in parentheses indicate the proportion of messages at each stage. All the results were statistically significant.

**Table 2:** Public engagement across different crisis stages and posting themes (1 January to 31 December 2020).

Theme	Likes			
	Prodromal Stage	Acute Stage	Chronic Stage	All Stages
Anti-epidemic	2.0 (11.7)	7.0 (36.6)	3.0 (17.5)	5.0 (28.0)
Solidarity	1.0 (6.2)	7.0 (37.6)	4.0 (19.1)	5.0 (28.7)
Hope	4.0 (161.5)	7.0 (39.3)	4.0 (22.7)	5.0 (29.2)
Gratitude	4.0 (10.3)	9.0 (62.3)	5.0 (23.2)	6.0 (41.3)
Optimism	0.0	10.0 (54.7)	5.5 (33.2)	8.0 (46.9)
Grit	0.0	5.0 (20.0)	3.0 (23.0)	4.0 (22.3)
All	3.0 (69.3)	7.0 (38.0)	4.0 (19.8)	5.0 (28.6)

Note: Values in the cells are medians; values in parentheses are means.

**Table 3:** Public engagement on Facebook postings with or without positive themes at different crisis stages in Macao (1 January to 31 December 2020).

Theme	Predictors Absent Median Likes (P25, P75)	Predictors Present Median Likes (P25, P75)	U Value	Z-Value	*p*-Value
Prodromal Stage					
Solidarity	9.500 (1.5, 44.8)	1.000 (0.0, 3.0)	118.500	−2.917	0.004
Acute Stage					
Gratitude	6.000 (2.0,21.0)	9.000 (2.0,32.0)	10,268,652.500	−7.721	<0.001
Hope	7.000 (2.0,22.0)	7.500 (2.0,24.0)	12,659,316.000	−2.366	0.018
Optimism	7.000 (2.0,22.0)	10.000 (2.0,36.0)	4,893,166.500	−7.040	<0.001
Chronic Stage					
Solidarity	3.000 (0.0, 15.0)	4.000 (1.0, 14.0)	18,024,253.500	−2.779	0.005
Anti-epidemic	5.000 (1.0, 18.0)	3.000 (0.0, 12.0)	17,946,686.000	−12.368	<0.001
Gratitude	3.000 (0.0, 13.0)	5.000 (1.0, 19.0)	12,328,662.500	−9.430	<0.001
Hope	3.000 (0.0, 14.0)	4.000 (1.0, 16.0)	17,957,420.500	−6.322	<0.001
Optimism	4.000 (0.0, 14.0)	5.500 (1.0, 23.0)	3,136,036.000	−5.371	<0.001

Note: Only significant variables are shown. Values in parentheses are numbers at the 25th percentile (P25) and 75th percentile (P75).

**Table 4:** Associations of content types, multimedia elements, message themes, and crisis stages with the level of likes (1 January to 31 December 2020).

Variables	Co-Efficient	Likes IRR	95% CI	Z-Value	*p*-Value
Intercept	1.945	6.993	6.212–7.872	32.185	<0.001
Content types					
Hyperlink	0.254	1.289	1.221–1.362	9.111	<0.001
Note	−0.283	0.754	0.597–0.951	−2.381	0.017
Status	0.219	1.245	1.174–1.321	7.292	<0.001
Multimedia Element					
Album	0.551	1.735	1.646–1.828	20.624	<0.001
Photograph	0.508	1.662	1.582–1.747	20.072	<0.001
Video	0.695	2.004	1.893–2.120	24.045	<0.001
Theme					
Solidarity	−0.014	0.986	0.960–1.012	−1.047	0.295
Grit	−0.140	0.870	0.805–0.940	−3.544	<0.001
Anti-epidemic	−0.108	0.897	0.872–0.924	−7.344	<0.001
Gratitude	0.453	1.573	1.524–1.623	28.044	<0.001
Hope	0.174	1.190	1.153–1.227	10.981	<0.001
Optimism	0.420	1.522	1.446–1.602	16.014	<0.001
Crisis Stage					
Prodromal	1.628	5.093	4.319–6.006	19.348	<0.001
Acute	0.542	1.719	1.605–1.841	15.476	<0.001
Chronic	0.139	1.149	1.097–1.202	5.940	<0.001

Note: IIR indicates incidence rate ratio, which was obtained by the exponent of coefficients.

## Data Availability

The data presented in this study are available on request from the author Xuechang Xian, at yb97322@um.edu.mo. The data are not publicly available in accordance with funding requirements and participant privacy.

## Supplementary Materials

The following supporting information can be downloaded at: https://www.mdpi.com/article/10.3390/ijerph19106159/s1, File S1: Examples of Positive Concepts in Facebook Posts with English Translation, File S2: (a) Keywords Used in the Study with English Translation; (b) Keywords Identified through Word2vec Word Embedding with Corresponding Similarity Scores, File S3: Message Themes across Crisis Stages of the Pandemic, Pairwise Comparison, File S4: Public Engagement across Different Crisis Stages, Pairwise Comparison.
