# Supplementary material for: Health Communication through Positive and Solidarity Messages Amid the COVID-19 Pandemic: Automated Content Analysis of Facebook Uses
Source: Int J Environ Res Public Health. 2022 May 19;19(10):6159. doi: 10.3390/ijerph19106159 (PMC9141526; doi:10.3390/ijerph19106159)
Supplement: Supplementary file 1 [file ijerph-19-06159-s001.zip › File S1. Examples of positive themes in Facebook posts with English translation.pdf]

File S1. Examples of positive themes in Facebook posts with English translation.

Theme	Definition	Example
Solidarity	Statement that advocates joint actions to contain the spread of the COVID-19	“讓我們一起響應特區政府呼籲，**團結**抗疫，給予仍在前線奮戰的“家人”最實在的支持！” “Let us respond to the appeal of the SAR government, **unite** in the fight against COVID-19, and give support to our "families" who are still fighting on the front lines.”
Anti-epidemic	Appeals to or motivate the public to fight against the COVID-19	“蓮城一心，無分你我，攜手**抗擊疫情**。” “Unite **against the COVID-19**.”
Gratitude	Statement that expresses gratitude and regards to those who have made contributions during the crisis	“**感恩**身處澳門這一福地，亦感激各位前線工作人員的奉獻” “I am grateful to be here in Macao, and we **thank** the medical staff at the frontline for their effort devoted to combat the virus.”
Hope	Statement that highlights the expectation or desire for good things to happen during the crisis	“**希望**疫情盡快過去，一切逐步回復正常吧 !” “I **hope** the epidemic will be over as soon as possible and everything will gradually return to normal.”
Optimism	Statement that provides positive thought and lift spirit of the public	“在大時代中我們疫境同行！澳門人**加油**！” “We are going together in the tough times! Macao people **come on! Add oil!**”
Grit	Mentions of perseverance to overcome difficulties	“面對逆境，正是對全民的**磨練**，只要反躬自省，一定能夠找到解決的辦法。” “**Difficult circumstances can temper one's will.** As long as we reflect on ourselves, we will be able to find a solution.”

Note: One message may have more than one concept.

Source links of examples:
1. Solidarity:
https://www.facebook.com/watch/?v=207990160599628

2. Anti-epidemic:
https://www.facebook.com/202772700178214/posts/857260701396074

3. Gratitude:
https://www.facebook.com/785937731445796/posts/3179939028712309

4. Hope:
https://www.facebook.com/349183078934/posts/10157906505723935

5. Optimism:
https://www.facebook.com/groups/1445500605709812/permalink/2683878415205352

6. Grit:
https://www.facebook.com/248547612010253/posts/1248802308651440
