# Supplementary material for: Health Communication through Positive and Solidarity Messages Amid the COVID-19 Pandemic: Automated Content Analysis of Facebook Uses
Source: Int J Environ Res Public Health. 2022 May 19;19(10):6159. doi: 10.3390/ijerph19106159 (PMC9141526; doi:10.3390/ijerph19106159)
Supplement: Supplementary file 1 [file ijerph-19-06159-s001.zip › File S2.pdf]

File S2a. Keywords used in the study with English translation.

1. Target words of COVID-19

"武漢不明原因肺炎 (Wuhan unknown pneumonia)" OR "新冠肺炎疫情 (the Coronavirus outbreak)" OR "武漢肺炎 (Wuhan pneumonia)" OR "新型冠狀病毒 (Novel Coronavirus)" OR "疫情 (pandemic)" OR "新冠疫情 (COVID-19 pandemic)" OR "抗疫 (anti-epidemic)" OR "防疫 (anti-epidemic)" OR "新冠病毒 (Novel Coronavirus)" OR "武漢病毒 (Wuhan virus)" OR " 中國病毒 (Chinese virus)" OR "功夫流感 (Kung Flu)" OR “中國肺炎 (China pneumonia)” OR “中國人肺炎 (Chinese pneumonia)” OR "COVID-19" OR "novel coronavirus" OR "2019-ncov" OR "NCP" OR "Novel Corona Pneumonia" OR "Chinese virus" OR "SARS-CoV-2" OR "pandemic" OR "epidemic" OR "Wuhan virus" OR "Wuhan pneumonia" OR "China pneumonia" OR "Chinese pneumonia"

2. Keywords for machine coding

(a) 團結 Solidarity

“團結 (solidarity)” OR “齊心協力 (make concerted effort)” OR “守望相助 (helping and supporting each other)” OR “同舟共濟 (the spirit of partnership)” OR “團結一心 (unite as one)” OR “相互支持 (mutual support)” OR “團結一致 (unite as one)” OR “團結互助 (work in unity and help one another)” OR “共同 (together)” OR “攜手 (hand in hand)” OR “同心協力 (unite in a concerted effort)” OR “攜手並肩 (Hand in hand side by side)” OR “凝聚 (hold together)” OR “並肩 (side by side)” OR “上下一心 (all unite as one)” OR “萬眾一心 (work with one heart)” OR “團結合作 (unite and cooperate)” OR “風雨同舟 (meet challenges together)” OR “眾志成城 (work as one)” OR “同心 (with one heart)” OR “患難與共 (face difficulties together)” OR “齊心合力 (make concerted efforts)” OR “攜手同心 (together we stand)” OR “心連心 (hearts closely linked together)” OR “共同努力 (concerted effort)” OR “通力合作 (pull together)” OR “共同合作 (mutual cooperation)” OR “風雨同路 (stand together) OR “共渡難關 (get over it)” OR “齊心 (stay in one mind)” OR “齊頭並進 (go hand in hand)” OR “團結抗疫 (fight epidemic with solidarity)” OR “一起抗疫 (fight epidemic together)” OR “協作 (collaborate)” OR “合力 (joint effort)”

(b) 抗疫 Anti-epidemic

“抗疫 (anti-epidemic)” OR “抗擊 (resist)” OR “戰疫 (fight the epidemic)” OR “抗緊疫 (anti-epidemic)” OR “抗疫長城 (a barrier against the epidemic)” OR “防疫長城 (a barrier for epidemic prevention)” OR “全民抗疫 (all the population together fight the epidemic)” OR “全城抗疫 (all the citizens together fight the epidemic)” OR “長期抗疫 (long term resistance to the epidemic)” OR “抗疫有你 (your involvement in the anti-epidemic career)”

(c) 希望 Hope

“希望 (hope)” OR “期望 (expect)” OR “冀望 (look forward to)” OR “期待 (expect)” OR “期盼 (anticipate)” OR “寄望 (place hope on)” OR “願 (wish)”

(d) 感謝 Gratitude

“感謝 (thanks)” OR “衷心感謝 (sincerely thank)” OR “非常感謝 (thank you so much)” OR “特別感謝 (special thanks)” OR “感激 (appreciate)” OR “多謝 (many thanks)” OR “表示感謝 (express gratitude)” OR “謝謝 (thank you)” OR “全賴 (owing to)” OR “無私奉獻 (selfless dedication)” OR “致謝 (express thanks)” OR “謝意 (thankfulness)” OR “報答 (pay back)” OR “貢獻 (contribution)” OR “答謝 (thank)” OR “作出貢獻 (make contribution)”

(e) 樂觀 Optimism
"加油 (keep it up)" OR "打氣 (encouragement)" OR "加油打氣 (go ahead)" OR "百毒不侵 (immune to diseases)" OR "鼓勁 (motivate)" OR "龍精虎猛 (vigorous)" OR "打打氣 (keep it up)"
(f) 勇敢 Grit
"勇敢 (courageous)" OR "奮鬥 (strive)" OR "堅韌 (tenacity)" OR "堅毅 (fortitude)" OR "逆境 (adversity)" OR "迎難而上 (overcome adversities)" OR "克服困難 (overcome difficulties)" OR "不畏艱險 (not afraid of the danger)" OR "逆流而上 (counter current)"

Note: Text in parenthesis indicates the meaning of the keyword in English.

File S2b. Keywords identified through Word2vec word embedding with corresponding similarity scores.

Grit 勇敢	***Solidarity*** 團結	***Anti-epidemic*** 抗疫	***Gratitude*** 感謝	***Hope*** 希望	***Optimism*** 加油
奮鬥 (strive) 0.587913275	齊心協力 (make concerted effort) 0.714468658	抗擊 (resist) 0.512936592	衷心感謝 (sincerely thank) 0.72669673	期望 (expect) 0.914320052	打氣 (encouragement) 0.645306349
堅毅 (fortitude) 0.541404724	守望相助 (helping and supporting each other) 0.707226157	抗緊疫 (anti-epidemic) 0.507600188	非常感謝 (thank you so much) 0.709448755	冀望 (look forward to) 0.682302296	加油打氣 (go ahead) 0.556103587
逆境 (adversity) 0.540107489	同舟共濟 (the spirit of partnership) 0.70074141	抗疫長城 (a barrier against the epidemic) 0.474552691	特別感謝 (special thanks) 0.704126	期待 (expect) 0.620945573	百毒不侵 (immune to diseases) 0.55442822
迎難而上 (overcome adversities) 0.539864779	團結一心 0.690964222 (unite as one)	防疫長城 (a barrier for epidemic prevention) 0.469383568	感激 (appreciate) 0.702687144	期盼 (anticipate) 0.598697066	鼓勁 (motivate) 0.538893461
克服困難 (overcome difficulties) 0.489892602	相互支持 (mutual support) 0.682557344	戰疫 (fight the epidemic) 0.468843609	多謝 (many thanks) 0.588089824	寄望 (place hope on) 0.560148358	龍精虎猛 (vigorous) 0.53035444
不畏艱險 (not afraid of the	團結一致 (unite as one)		表示感謝 (express		打打氣 (keep it up)

danger) 0.467252612	0.661968589		gratitude) 0.578718543		0.503768146
逆流而上 (counter current) 0.463056326	團結互助 (work in unity and help one another) 0.647435546		謝謝 (thank you) 0.56649065		
	共同 (together) 0.639477849		全賴 (owing to) 0.543093503		
	攜手 (hand in hand) 0.636185408		無私奉獻 (selfless dedication) 0.523185313		
	同心協力 (unite in a concerted effort) 0.632137835		致謝 (express thanks) 0.519904971		
	攜手並肩 (hand in hand side by side) 0.619895577		謝意 (thankfulness) 0.502565205		
	凝聚 (hold together) 0.617751837		報答 (pay back) 0.489438593		
	並肩 (side by side) 0.602886021		貢獻 (contribution) 0.4877159		
	上下一心 (all unite as one) 0.596060634		答謝 (thank) 0.487063736		
	萬眾一心 (work with one heart) 0.595138311		作出貢獻 (make contribution) 0.486494362		
	團結合作 (unite and cooperate) 0.587534547				
	風雨同舟 (meet challenges together) 0.583126426				
	眾志成城				

	(work as one) 0.571036637				
	同心 (with one heart) 0.542360067				
	患難與共 (face difficulties together) 0.529419303				
	齊心合力 (make concerted efforts) 0.523750305				
	攜手同心 (together we stand) 0.519810677				
	心連心 (hearts closely linked together) 0.507393599				
	共同努力 (concerted effort) 0.504318058				
	通力合作 (pull together) 0.500597477				
	風雨同路 (meet challenges together) 0.491230011				
	共渡難關 (get over it) 0.489358038				

Note: Terms in the table headers represent meanings of the six themes and were used as the test words in the Word2vec model, while those in the cells are corresponding synonyms with cosine similarity scores calculated. Text in parenthesis indicates the meaning of the keyword in English.
