# Supplementary material for: Health Communication through Positive and Solidarity Messages Amid the COVID-19 Pandemic: Automated Content Analysis of Facebook Uses
Source: Int J Environ Res Public Health. 2022 May 19;19(10):6159. doi: 10.3390/ijerph19106159 (PMC9141526; doi:10.3390/ijerph19106159)
Supplement: Supplementary file 1 [file ijerph-19-06159-s001.zip › File S3. Message themes across crisis stages of the pandemic, pairwise comparison..pdf]

File S3. Posting themes across crisis stages of the pandemic, pairwise comparison.

Theme* Crisis Stage Crosstabulation

			Crisis Stage			
			Acute stage	Chronic stage	Prodromal stage	Total
Theme	Anti-epidemic	Count	7779_{a}	6429_{b}	11_{b}	14219
		% within Attribute	54.7%	45.2%	0.1%	100.0%
	Gratitude	Count	2399_{a}	2781_{b}	$6_{a, b}$	5186
		% within Attribute	46.3%	53.6%	0.1%	100.0%
	Hope	Count	2883_{a}	4798_{b}	17_{b}	7698
		% within Attribute	37.5%	62.3%	0.2%	100.0%
	Optimism	Count	1035_{a}	590_{b}	$1_{a, b}$	1626
		% within Attribute	63.7%	36.3%	0.1%	100.0%
	Resilience	Count	176_{a}	539_{b}	$0_{a, b}$	715
		% within Attribute	24.6%	75.4%	0.0%	100.0%
	Solidarity	Count	4870_{a}	4430_{b}	24_{c}	9324
		% within Attribute	52.2%	47.5%	0.3%	100.0%
Total		Count	19142	19567	59	38768
		% within Attribute	49.4%	50.5%	0.2%	100.0%

Each subscript letter denotes a subset of crisis stage categories whose column proportions do not differ significantly from each other at the 0.05 level if they are annotated with a similar subscript. Reversely, within each row, percentages that don't share a subscript are significantly different. Tests are adjusted for all pairwise comparisons using the Bonferroni correction.
