# Supplementary material for: Health Communication through Positive and Solidarity Messages Amid the COVID-19 Pandemic: Automated Content Analysis of Facebook Uses
Source: Int J Environ Res Public Health. 2022 May 19;19(10):6159. doi: 10.3390/ijerph19106159 (PMC9141526; doi:10.3390/ijerph19106159)
Supplement: Supplementary file 1 [file ijerph-19-06159-s001.zip › File S4. Public engagement across different crisis stages, pairwise comparison..pdf]

File S4. Public engagement across different crisis stages, pairwise comparison.

	Likes			
	**Test statistic**	**Std. test statistic**	**Sig.**	**Adj. sig.**
**Prodromal - Acute**	-2,676.386	-2.490	0.013	0.038
**Prodromal - Chronic**	-543.086	-0.505	0.613	1.000
**Acute - Chronic**	2,133.301	23.561	<0.001	<.001
